# Posttranscriptional Regulation Controls Calretinin Expression in Malignant Pleural Mesothelioma

**DOI:** 10.3389/fgene.2017.00070

**Published:** 2017-05-29

**Authors:** Jelena Kresoja-Rakic, Merve Sulemani, Michaela B. Kirschner, Manuel Ronner, Glen Reid, Steven Kao, Beat Schwaller, Walter Weder, Rolf A. Stahel, Emanuela Felley-Bosco

**Affiliations:** ^1^Laboratory of Molecular Oncology, Division of Thoracic SurgeryUniversity Hospital Zurich, Switzerland; ^2^Division of Thoracic SurgeryUniversity Hospital Zurich, Switzerland; ^3^Asbestos Diseases Research Institute, SydneyNSW, Australia; ^4^School of Medicine, The University of Sydney, SydneyNSW, Australia; ^5^Department of Medical Oncology, Chris O’Brien Lifehouse, SydneyNSW, Australia; ^6^Department of Medicine, Anatomy, University of FribourgFribourg, Switzerland; ^7^Clinic for OncologyUniversity Hospital Zurich, Zurich

**Keywords:** cancer, 3′ untranslated region, microRNA, non-coding RNA, mesothelioma, calretinin

## Abstract

Calretinin (CALB2) is a diagnostic and prognostic marker in malignant pleural mesothelioma (MPM). We previously reported that calretinin expression is regulated at the mRNA level. The presence of a medium-sized (573 nucleotide) 3′ untranslated region (3′UTR) predicted to contain binding sites for miR-30a/b/c/d/e and miR-9 as well as an adenine/uridine-rich element (ARE) in all three transcripts arising from the *CALB2* gene, suggests that calretinin expression is regulated via posttranscriptional mechanisms. Our aim was to investigate the role of the CALB2-3′UTR in the posttranscriptional regulation of calretinin expression in MPM. CALB2-3′UTR was inserted downstream of the luciferase reporter gene using pmiRGLO vector and reporter expression was determined after transfection into MPM cells. Targeted mutagenesis was used to generate variants harboring mutated miR-30 family and ARE binding sites. Electrophoretic mobility shift assay was used to test for the presence of ARE binding proteins. CALB2-3′UTR significantly decreased luciferase activity in MPM cells. Analysis of mutation in the ARE site revealed a further destabilization of the reporter and human antigen R (HuR) binding to the ARE sequence was detected. The mutation of two miR-30 binding sites abolished CALB2-3′UTR destabilization effect; a transient delivery of miR-30e-5p mimics or anti-miR into MPM cells resulted in a significant decrease/increase of the luciferase reporter expression and calretinin protein, respectively. Moreover, overexpression of CALB2-3′UTR quenched the effect of miR-30e-5p mimics on calretinin protein levels, possibly by sequestering the mimics, thereby suggesting a competitive endogenous RNA network. Finally, by data mining we observed that expression of miR-30e-5p was negatively correlated with the calretinin expression in a cohort of MPM patient samples. Our data show the role of (1) adenine-uridine (AU)-binding proteins in calretinin stabilization and (2) miR-30e-5p in the posttranscriptional negative regulation of calretinin expression via interaction with its 3′UTR. Furthermore, our study demonstrates a possible physiological role of calretinin’s alternatively spliced transcripts.

## Introduction

Malignant pleural mesothelioma (MPM) is an aggressive form of cancer arising from mesothelial cells lining the pleural cavity and is mainly resulting from the inhalation of asbestos fibers (Porpodis et al., 2013). Calretinin, a Ca^2+^ binding protein, is a diagnostic and prognostic marker for mesothelioma (Kao et al., 2011; Linton et al., 2014; Ordóñez, 2014; Otterstrom et al., 2014). So far, molecular mechanisms driving calretinin expression in mesothelioma remain largely unknown. We have recently described that the expression of calretinin is regulated at the level of transcription by nuclear respiratory factor 1 (NRF-1) and E2F2 transcription factors (Kresoja-Rakic et al., 2016). As the *CALB2* mRNA includes a so-called medium size (Fanourgakis et al., 2016) 3′ untranslated region (3′UTR) (573 bp) containing an adenine/uridine-rich element (ARE) motif as well as a microRNA (miRNAs) binding sequences, this suggests that posttranscriptional mechanisms of regulation are also involved.

The ARE motif is the most frequently studied *cis*-acting element that plays a role in the posttranscriptional control of gene expression by affecting mRNA stability. Its dysregulation has also been described in cancer (Khabar, 2010). The ARE-containing mRNAs are directly or indirectly bound by *trans*-acting RNA-binding proteins (RBPs) known as adenine-uridine (AU)-binding proteins (AUBPs), which can in turn promote degradation, deadenylation (ARE-mediated decay—AMD) or even stabilization of mRNA. There are 21 AUBPs implicated in either stabilization or destabilization of targeted ARE-mRNA (von Roretz et al., 2011). The most extensively studied AUBPs are HuR/ELAVL1 (human antigen R, embryonic lethal abnormal vision, Drosophila—stabilization; Ma et al., 1996; Brennan and Steitz, 2001), TTP (tristetraprolin), and AUF (ARE/poly(U)-binding/degradation factor 1—destabilization) (Barreau et al., 2005).

miRNAs are small (18–22 nt) non-coding RNAs that affect gene expression on the posttranscriptional level. miRNAs bind to partially complementary miRNA recognition elements (MREs) within targeted mRNA, leading to inhibition of translation or mRNA destabilization (Krol et al., 2010). In general, there is a global decrease of miRNA expression in MPM in concordance with other cancer types (Lu et al., 2005; Volinia et al., 2006; Reid, 2015). The TargetScan (Agarwal et al., 2015), miRanda (Betel et al., 2008), and PicTar (Krek et al., 2005) databases predict potential binding sites for miR-30a/b/c/d/e and miR-9 within the calretinin 3′UTR. Based on the analysis of 1319 differentially expressed genes, Cheng et al. (2016) identified the miR-30 family amongst the top 20 enriched miRNA families in mesothelioma. Furthermore, miR-30e-5p is a part of the 6-miRNA signature shown to predict long survival in mesothelioma patients (Kirschner et al., 2015). So far there was no study on the biological function of the miR-30 family or AUBP in mesothelioma.

An additional level of complexity is achieved through the interaction (antagonistic or cooperative) between miRNA and RBPs (von Roretz and Gallouzi, 2008; van Kouwenhove et al., 2011). Therefore, 3′UTR can mediate posttranscriptional gene expression regulation, acting as a platform for the individual effect or the crosstalk between miRNA or AUBP.

An indirect evidence for a functional role of the CALB2-3′UTR has been suggested in a study using *Calb2*-IRES-Cre transgene, in which Cre recombinase was inserted into the 3′UTR of the mouse *Calb2* gene (Tasic et al., 2016), apparently disrupting regulatory elements and leading to discrepancy between Cre expression and endogenous calretinin expression in adult mice. Here, using a reporter system and mutational analysis of the predicted putative *cis*-regulatory sites in the CALB2-3′UTR followed by overexpression or inhibition, we demonstrated that miR-30e-5p is able of modulating calretinin expression. Additionally, we show that the ARE element in the 3′UTR stabilizes *CALB2* mRNA. Furthermore, we suggest a role for alternative transcripts of the calretinin gene, where their 3′UTRs might compete for the pool of *trans*-acting factors, thus affecting calretinin expression. All in all, this data may explain the highest efficiency of targeting 3′ sequence for silencing calretinin (Blum et al., 2017) which has been suggested as a potential therapeutic strategy for epithelioid MPM (Blum and Schwaller, 2013).

## Materials and Methods

### Cell Lines

Four mesothelioma cell lines were used in experiments. ACC-MESO-4 cell line was obtained from RIKEN BioResource Centre (Usami et al., 2006) and maintained in RPMI-1640 medium (Sigma-Aldrich) supplemented with 15% fetal calf serum (FCS), 1% penicillin/streptomycin (P/S) and 2 mM L-glutamine. Both ZL55 and SPC111 cell lines were established in our lab (Schmitter et al., 1992) whereas ONE58 was obtained from the European Collection of Cell Cultures (Salisbury, United Kingdom; Manning et al., 1991). ZL55 cells were cultured as described by Thurneysen et al. (2009). SPC111 and ONE58 cells were maintained in DMEM/F12 supplemented with 15% FCS, 1% P/S and 2 mM L-glutamine.

### Generation of the CALB2-3′UTR and Mutant Luciferase Reporters

To generate the luciferase reporter construct pmiRGLO-CALB2-3′UTR (72800 Addgene), CALB2-3′UTR was amplified from genomic DNA (ZL55) using the following tailed primers: forward *Nhe*I 5′-ATTTGCTAGC AGTGGGGACGGGGGCTGCTT-3′, and reverse *Sal*I 5′-ACGTGTCGACGGGTAAGTTTCCACAGCAGG-3′. The polymerase chain reaction (PCR) was performed in a total volume of 50 μl containing 1× Colorless Go Taq^®^ Flexi Buffer (Promega), 2 mM MgCl_2_ solution, 0.2 mM PCR Nucleotide Mix, 0.2 μM of each primer, 1.25 U of Go Taq^®^ G2 Hot Start Polymerase (Promega) and 25 ng of genomic DNA. The sequence of interest was amplified by means of touchdown PCR system: denaturation at 95°C, annealing at 60°C/63°C/65°C/68°C, extension at 74°C (10 cycles for each different annealing temperature condition). Amplified CALB2-3′UTR was cloned downstream of the Firefly luciferase coding sequence in the pmiRGLO vector (Cat. No. 1330, Promega). The QuickChange Site-Directed mutagenesis kit (200518, Agilent technologies) was employed to introduce mutations in the ARE or miR-30 sites with the following primers: pmiRGLO-CALB2-3′UTRmtARE (74425 Addgene), 5′-ctctgttggacatagaagcccagaccatacagcgagggagctcat-3′, 5′-atgagctccctcgctgtatggtctgggcttctatgtccaacagag-3′; pmiRGLO-CALB2-3′UTRmir30mt (74428 Addgene), 5′-cgtgctccttttctctttgggtttcttttatcccaaagaagagtttacagacaat-3′, 5′-attgtctgtaaactcttctttgggataaaagaaacccaaagagaaaaggagcacg-3′; pmiRGLO-CALB2-3′UTRmir30dmt (74429 Addgene), 5′-ttgggtttcttttatcccaaagaagattatccagacaataaaatggaaaggtcctgc-3′, 5′-gcaggacctttccattttattgtctggataatcttctttgggataaaagaaacccaa-3′ and, a combination of primers above to construct pmiRGLO-CALB2-3′UTR-mir30dmt-mtARE (74430 Addgene).

### Stable and Transient Transfection, and Luciferase Reporter Assay

To generate stable cell lines carrying pmiRGLO-*CALB2*-3′UTR or empty pmiRGLO, 1 × 10^5^ ACC-Meso-4 or ONE58 cells were transfected with 200 ng of the corresponding plasmid and 1 μl of Lipofectamine 2000 in six-well plate. On the following day, geneticin-containing medium was added and replaced every 3–4 days until cells reached confluence for a further expansion.

For transient plasmid transfection, 0.8–1 × 10^5^ cells were seeded in 12-well plate. On the following day, 200 ng plasmid along with 1 μl of DMRIE-C transfection reagent mixed in 800 μl of OPTIMEM, was added to the corresponding wells and incubated for 9 h. After 48 h, transfected cells were lysed and reporter activity was measured afterward using the Dual-Luciferase reporter assay according to manufactures instructions (Promega, Madison, WI, United States).

### miRNA Mimic and Inhibitor Treatment

For mimics and anti-miR treatment, following mimics were used: 1 or 5 nM of has-miR-30b-5p (MSY0000420, Qiagen), has-miR-30c-5p (MSY0000244, Qiagen), hsa-miR-9-5p (MSY0000441, Qiagen), hsa-miR-30e-5p (Shanghai GenePharma Co., Ltd), or 10 nM or 30 nM anti-hsa-miR-30e-5p (MIN0000692, Qiagen), respectively, was transiently delivered using Lipofectamine RNAiMAX according to the manufacturer’s reverse transfection protocol as previously described (Shi et al., 2012). 5 × 10^4^ cells were then plated in 12-wells plate and whole cell lysates were prepared after 72 h for the luciferase or protein measurement.

### Relative Gene Expression and Western Blotting

RNA was extracted and cDNA was prepared as previously described (Sidi et al., 2011). Relative mRNA levels were determined by comparing the PCR cycle thresholds between cDNA of a specific gene and histone (ΔCt method) (Andre and Felley-Bosco, 2003). Selected gene expression analysis using Minimum Information on Quantitative Experiments (MIQE) compliant protocols was conducted as previously described (Bustin et al., 2009). Following genes were quantified: HuR (5′-GGGCTATGGCTTTGTGAACTA-3′; 5′-GCGAGCATACGACACCTTAAT-3′); TTP (5′-GGATCCGACCCTGATGAATATG-3′; 5′-GAAACAGAGATGCGATTGAAGATG-3′; Firefly luciferase (5′-GTGGTGTGCAGCGAGAATAG-3′; 5′-CGCTCGTTGTAGATGTCGTTAG-3′); Renilla luciferase (5′-CGTTGGCTACCCGTGATATT-3′; 5′-CTCGTCAAGAAGGCGATAGAAG-3′), primers detecting *CALB2* alternative transcripts are listed in Supplementary Figure 1.

miRNA expression was determined according manufacturer’s protocols using the miRNeasy Mini Kit (Qiagen), miScript II RT (Qiagen), miScript primer assays (Qiagen), and miScript SYBR Green PCR Kit (Qiagen) in the 7900HT Fast Real-Time PCR System (SDS, ABI/Perkin Elmer). Relative miRNA levels were determined by comparing the PCR cycle thresholds between cDNA of a specific gene and RNU-6B-2 (ΔCt method).

Calretinin protein expression was analyzed as previously described (Kresoja-Rakic et al., 2016).

### Electrophoretic Mobility Shift Assay (EMSA) for RNA–Protein Complexes

Cytosolic protein extracts were isolated using the NE-PER^TM^ Nuclear and Cytoplasmic Extraction Kit (78833, Pierce Biotechnology) according to manufacturer’s instructions. To demonstrate possible interactions of RBP and single stranded RNA containing AUUUA pentamer, RNA-EMSA was performed using LightShift^®^ Chemiluminescent RNA EMSA Kit (Pierce Biotechnology). The binding reaction (20 μl total) contained 1× binding buffer (10 mM HEPES, pH 7.3), 20 mM KCL, 1 mM MgCl2, 1 mM DTT), 2.5% glycerol, 100 mM KCl, 2.5 μg heparin (Sigma-Aldrich), 5 μg of cytosolic protein extract. After 5 min incubation on ice, 40 fmol specific biotinylated RNA oligonucleotides (5′-UCGCUGUAUGAUUUAGGCUUCUAUG-3′) was added. For competition reactions, 200-fold excess of either specific or unrelated unlabeled RNA oligo probes (5′-UCCUGCUUCAACAGUGCUUGGACGGAAC-3′) were incubated with binding reactions for 5 min prior to addition of specific biotinylated RNA oligo probe. The reactions were incubated for 25 min at room temperature and then separated on a 6% polyacrylamide 0.5× Tris Borate EDTA (TBE) native gel. After the transfer on a nylon membrane, RNA probe–protein complex was visualized using Chemiluminescent Nucleic Acid Detection Module Kit Module (89880, Thermo Scientific Pierce) according to manufacturer’s protocol.

### RNA–Protein Immunoprecipitation

Complete magnetic RNA-protein immunoprecipitation was performed according to the protocol of Pierce Magnetic RNA-Protein Pull-Down kit (Thermo Scientific, 20164) using 50 pmol of RNA and 75 μg of native cytoplasmic protein fraction of ACC-MESO-4 cells.

### Relationship between miR-30e-5p and Calretinin in Tumor Samples

We datamined previous studies where both calretinin immunohistochemistry (IHC) evaluation (Kao et al., 2011) and miR-30e-5p (Kirschner et al., 2015) had been determined in tumor samples from mesothelioma patients who underwent extrapleural pneumonectomy between 1994 and 2009 at Royal Prince Alfred and Strathfield Private Hospital, Sydney, Australia. Correlation between calretinin and miR-30e-5p expression was analyzed in a subset of 60 tumor samples, for which both measurements were available (Supplementary Table 1).

### Statistics

Data are expressed as mean ± standard deviation of multiple experiments. Statistical analysis was performed using Mann–Whitney *U*-tests using StatView 5.0.1 (SAS Institute) and *t*-test using GraphPad. Spearman’s correlation test was used to calretinin tumor positive cells (%) vs. miR-30e-5p relative expression levels. Differences were considered statistically significant at *p* < 0.05.

Calretinin 3′UTR contains a stabilizing and destabilizing elements. **(A)** Multiple species alignment of calretinin 3′UTR using UCSC Genome Browser revealed two conserved stretches. **(B)** Complete calretinin 3′UTR sequence 573 nt (CALB2-3′UTR) with the predicted putative *cis*-regulatory elements: an adenine/uridine-rich motif (ARE) (red), miR-30a/b/c/d/e (red), miR-9 (blue) binding sites and a poly-A site (PAS) (green). **(C)** 573-nt CALB2-3′UTR was inserted downstream of the luciferase reporter in the pmiRGLO reporter plasmid. Four additional luciferase reporter constructs carrying mutations in either ARE and/or miR-30 binding sites were generated and their activity was tested in ONE58 cells. **(D)** Insertion of CALB2-3′UTR significantly downregulated luciferase expression when compared to the vector pmiRGLO alone. Mutation of the ARE-motif further downregulated reporter expression, whereas the double mutation of miR-30 binding sites abolished the downregulatory effect of the calretinin 3′UTR. FLU—Firefly Luciferase Units. The empty vector pmiRGLO plasmid expression was arbitrarily set to 100% and the reporter activity of the mutants was expressed as a percentage of the wild-type construct. Mean ± SD, *n* = 8; ^∗^*p* < 0.05; ^∗∗^*p* < 0.01; ^∗∗∗^*p* < 0.005 using Mann–Whitney *U*-test.

## Results

### Calretinin 3′UTR Harbors a Functional ARE Motif and miR-30 Sites

**FIGURE 1:**
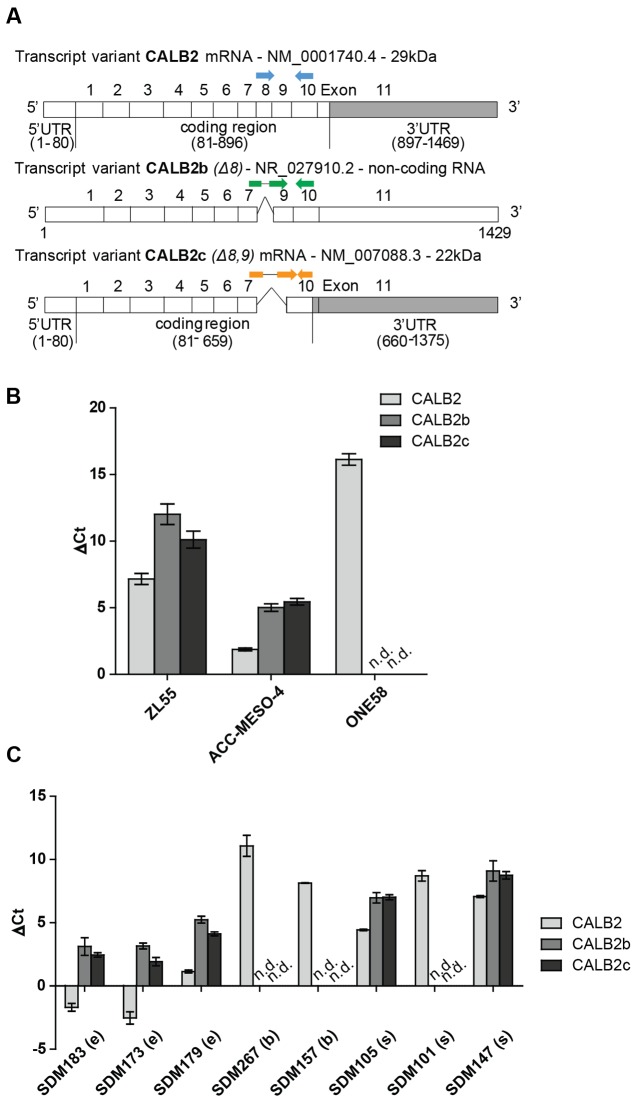
Three different calretinin transcripts are present in mesothelioma cells. **(A)** The calretinin gene (*CALB2*) has been reported to have a normally spliced transcript (full isoform CALB2-29 kDa) with 11 exons, and two alternative splice variants CALB2b (non-coding RNA) and CALB2c (short isoform 22 kDa) with skipped exon 8 and exons 8, 9, respectively. Labeled in gray box is the 3′UTR (untranslated region) of *CALB2* mRNA variants. Arrows in blue, green, and orange, depict the positions of boundary spanning primers used to detect the presence of calretinin transcripts. **(B)** RT-qPCR revealed the presence of all three transcripts in ZL55 and ACC-MESO-4 cells whereas only full isoform CALB2 (normal spliced transcript) and at low abundance is detected in ONE58 cells. **(C)** The expression profile of all three calretinin transcripts is detectable in a panel of mesothelioma tumors (e, epithelioid; b, biphasic; s, sarcomatoid; n.d., not detected). Histones were used as internal control.

The calretinin gene (*CALB2*), located on chromosome 16, has three different transcripts: (1) the full-length isoform 1 encoding the 29 kDa protein—CALB2; (2) a non-coding alternatively spliced variant—CALB2b (Δ8); and (3) an alternatively spliced variant giving rise to a 22k Da calretinin protein isoform—CALB2c (Δ8, 9) (**Figure 1A**; Schwaller et al., 1995). The length of the 3′UTR is 573 and 716 nt in CALB2 and CALB2c, respectively. The alternative transcript CALB2b contains the same sequence of the 573 nt 3′UTR but the whole transcript is considered as a non-coding since the use of the 5′-most supported translation start codon introduces a premature termination codon rendering the transcript a candidate for nonsense-mediated mRNA decay.

To detect the presence of calretinin variants CALB2, CALB2b, and CALB2c, real-time quantitative PCR (RT-qPCR) was performed with cDNA from ZL55, ACC-MESO-4 and ONE58 cells using boundary-spanning primers approach (**Figure 1A** and Supplementary Figure 1). The highest level of all three transcripts was detected in ACC-MESO-4 cells followed by lower, but still detectable, expression of all three transcripts in ZL55 cells, whereas only the full-length isoform CALB2 was detected in ONE58 cells (**Figure 1B**). At the protein level, only the full-length 29 kDa calretinin was detected in all three cells lines, ACC-MESO-4 (high-calretinin), ZL55 (intermediate calretinin), and ONE58 (low-calretinin), as reported in a previous study (Kresoja-Rakic et al., 2016). Similar to ONE58 cells, we detected only the full-length transcript at low levels in SPC111 (low-calretinin expressing) cells (data not shown). Analysis of tumor samples confirmed that the expression profile of the three transcripts observed in cell lines reflects splicing events that are also present in tumors, with the full-length CALB2 being in all cases the most abundant transcript (**Figure 1C**).

**FIGURE 2:**
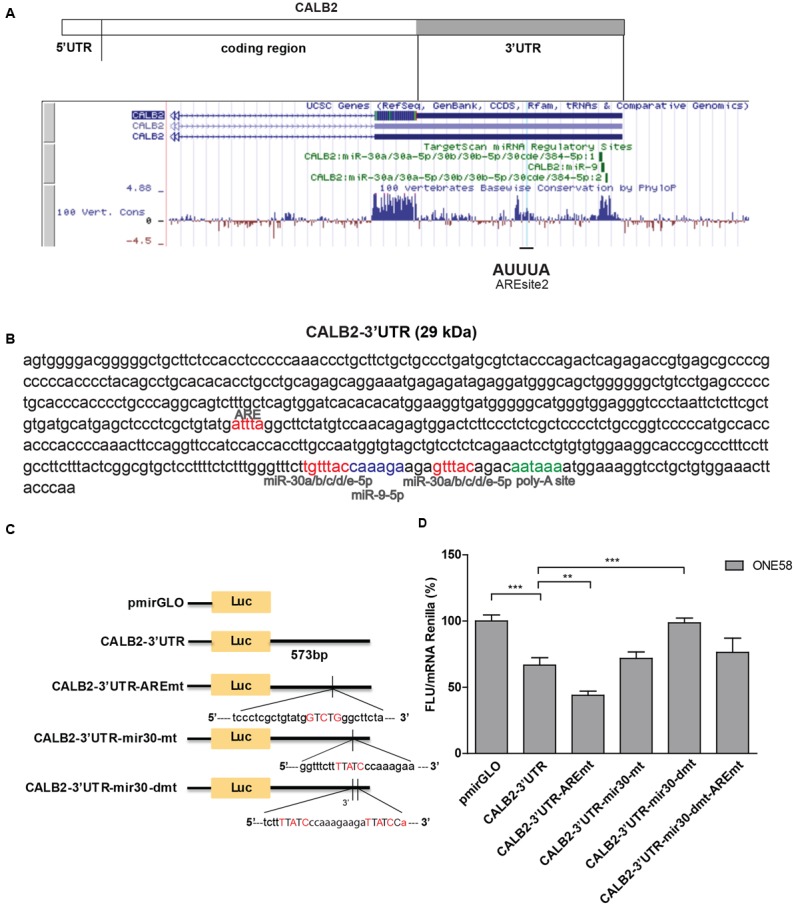
Calretinin 3′UTR contains a stabilizing and destabilizing elements. **(A)** Multiple species alignment of calretinin 3′UTR using UCSC Genome Browser revealed two conserved stretches. **(B)** Complete calretinin 3′UTR sequence 573 nt (CALB2-3′UTR) with the predicted putative *cis*-regulatory elements: an adenine/uridine-rich motif (ARE) (red), miR-30a/b/c/d/e (red), miR-9 (blue) binding sites and a poly-A site (PAS) (green). **(C)** 573-nt CALB2-3′UTR was inserted downstream of the luciferase reporter in the pmiRGLO reporter plasmid. Four additional luciferase reporter constructs carrying mutations in either ARE and/or miR-30 binding sites were generated and their activity was tested in ONE58 cells. **(D)** Insertion of CALB2-3′UTR significantly downregulated luciferase expression when compared to the vector pmiRGLO alone. Mutation of the ARE-motif further downregulated reporter expression, whereas the double mutation of miR-30 binding sites abolished the downregulatory effect of the calretinin 3′UTR. FLU—Firefly Luciferase Units. The empty vector pmiRGLO plasmid expression was arbitrarily set to 100% and the reporter activity of the mutants was expressed as a percentage of the wild-type construct. Mean ± SD, *n* = 8; ^∗^*p* < 0.05; ^∗∗^*p* < 0.01; ^∗∗∗^*p* < 0.005 using Mann–Whitney *U*-test.

The complete 3′UTR sequence of the full-length calretinin isoform CALB2, was used in further experiments (CALB2-3′UTR). Multiple sequence alignment of CALB2-3′UTR across 100 vertebrates listed in the UCSC Genome Browser, indicated two conserved stretches within the CALB2-3′UTR (**Figure 2A**). The AREsite2 software (Fallmann et al., 2016) revealed a putative ARE motif (AUUUA) within the first stretch, and TargetScan7.1 software predicted two binding sites for the miR-30 family and one for miR-9, within the second conserved stretch (**Figures 2A,B**).

In order to test its functionality, CALB2-3′UTR was inserted downstream of the Firefly luciferase reporter gene (pmiRGLO vector) and transiently transfected into ONE58 cells (**Figure 2C**). Insertion of the CALB2-3′UTR led to 40% (*p* = 0.0012) decreased expression of Firefly luciferase when compared to the empty vector (pmiRGLO), indicating that the calretinin 3′UTR conveys a downregulatory effect (**Figure 2D**). We then generated an additional four constructs harboring mutations of the consensus sequence for the predicted ARE motif and miR-30 binding sites and transiently transfected all variants into ONE58 cells to test for their effects on luciferase expression (**Figures 2C,D**). Mutation of the predicted ARE motif induced an even further significant decrease of 20% (*p* = 0.0055), indicating that the ARE site binds to a stabilizing factor. In contrast, mutation of both miR-30 binding sites restored the expression of the luciferase reporter to the level of the empty vector. This downregulatory effect is possibly conveyed through the second miR-30 binding site alone. Despite a predicted miR-9 binding site, mimics treatment did not exert downregulatory effect on the reporter or calretinin expression (Supplementary Figure 2) thus excluding this miR for further mutational analysis. Taken together, our data indicate that the 3′UTR of the *CALB2* mRNA contains multiple elements contributing to posttranscriptional regulation and control of calretinin protein levels.

### Specific Binding of a Protein to the 25 nt-Sequence Containing ARE-Motif

Having observed that the ARE motif in the calretinin 3′UTR is functional, we investigated the possible interaction between this site and a potential stabilizing *trans*-acting factor. Indeed, investigation of the optimal secondary structure of the *CALB2*-3′UTR sequence (317 nt; 1152—1469 nt) using the RNAfold prediction tool (Gruber et al., 2008), revealed a secondary structure in which the ARE site forms a bulge and thus might be accessible to an interactor. The reason for using a stretch of 317 nt containing the predicted regulatory sequences was to increase accuracy and to provide a better overview (Hofacker, 2009). Replacing the AUUUA with GUCUG results in a mutated ARE motif paired in a newly formed secondary structure predicted to be less likely to be accessible for putative interacting protein (Supplementary Figure 3).

**FIGURE 3:**
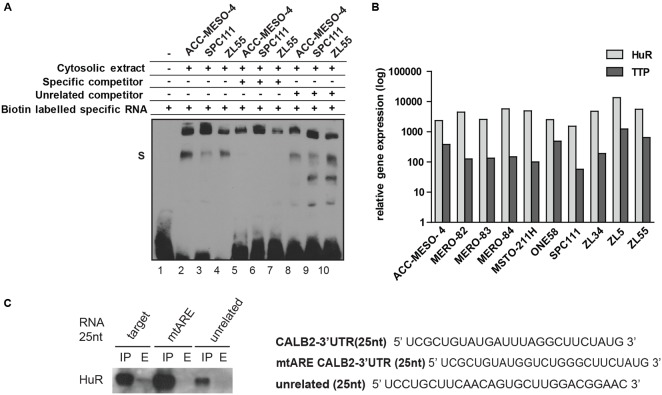
25 nt RNA oligo containing ARE-motif binds a protein from cytosolic extract of mesothelioma cell lines. **(A)** EMSA assay demonstrating that the 25-nt RNA oligo containing the ARE element binds a protein present in cytosolic protein extracts of ACC-MESO-4, SPC111, and ZL55 cells (lanes 2–4). The complex is diminished in the presence of a 200-folds excess of the same unlabeled probe (lanes 5–7), but not outcompeted in a 200-folds excess of the unrelated unlabeled probe (lanes 8–10). **(B)** Quantification of two AU-binding proteins, HuR and TTP, across different mesothelioma cell lines, using histones as an internal control. Levels are shown relative to the TTP in MSTO-211H cells according to the –ΔΔCt method. **(C)** Western blot detection of HuR following RNA–protein pull-down assay that was carried out using 75 μg of cytosolic proteins with 50 pmol of desthiobiotin-RNA used in EMSA assay.

We then employed a biochemical approach using electrophoretic mobility shift assay to demonstrate the specific interaction between a 25 nt RNA containing the ARE motif (UCGCUGUAUGAUUUAGGCUUCUAUG) and a cytosolic extract containing RBPs (**Figure 3A**). Incubation of the biotin-labeled 25 nt RNA with a cytosolic protein extract of ACC-MESO-4, ZL55, and SPC111 cells showed a protein–RNA complex formation (**Figure 3A**, lanes 2–4). To further confirm the specificity of the RNA–protein interaction, competing reactions were performed using a 200-fold excess of the identical 25 nt unlabeled sequence RNA (specific competitor) or unlabeled-unrelated RNA-oligo (unspecific competitor). Addition of the specific competitor decreased the detectable RNA–protein complex (**Figure 3A**, lanes 5–7), whereas the excess of the non-specific competitor did not outcompete the biotin–RNA–protein complex (**Figure 3A**, lanes 8–10). The presence of the additional lower bands in the lanes 9 and 10 are assumed to be different secondary structures formed by the biotin-RNA oligo.

Since the mutational reporter analysis had identified the ARE motif as a stabilizing element, we quantified the expression of HuR (stabilizer) along with TTP (destabilizer) across 10 mesothelioma cell lines and observed that HuR is abundantly expressed in all tested cells lines and 5- to 50-fold enriched compared to TTP (**Figure 3B**). We confirmed overexpression of HuR in mesothelioma compared to normal pleura along with a low TTP/HuR ratio by investigating microarray data (Gordon et al., 2005). In addition, a 25 nt-biotinylated RNA with an ARE motif, but not the mutated RNA oligo or an unspecific sequence, pulled-down HuR (**Figure 3C**). These results demonstrated that some of the mostly studied AUBP (HuR, TTP) are expressed in mesothelioma cells and that the 25 nt-fragment of *CALB2*-3′UTR harboring the ARE element binds HuR.

### miR-30e-5p Downregulates Calretinin Protein Expression

**FIGURE 4:**
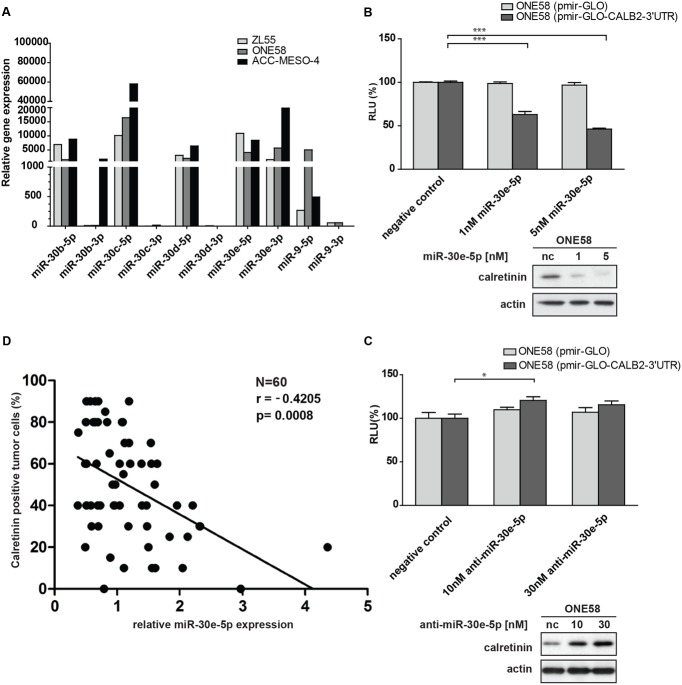
Members of miR-30 family are abundantly expressed in mesothelioma cell lines and miR-30e-5p regulates calretinin expression. **(A)** miR-30e-5p mimic treatment significantly repressed reporter expression in ONE58 cells stable expressing reporter-CALB2-3′UTR along with downregulation of calretinin protein levels. Mean ± SD, *n* = 6; ^∗^*p* < 0.05; ^∗∗^*p* < 0.01; ^∗∗∗^*p* < 0.005 using Mann–Whitney *U*-test. **(B)** Depletion of miR-30e-5p by anti-miR-30e-5p increased the reporter expression as well as calretinin protein levels. Mean ± SD, *n* = 3; ^∗^*p* < 0.05; ^∗∗^*p* < 0.01; ^∗∗∗^*p* < 0.005 using *t*-test. **(C)** Quantitative RT-PCR analysis of miR-30 family members and miR-9 in three mesothelioma cell lines using as RNU-6B-2 as a reference gene. Levels are shown relative to the miR-30d-5p in ZL55 cells according to the –ΔΔCt method. ^∗^*p* < 0.005. **(D)** Weak negative correlation between calretinin expression (IHC) and miR-30e-5p in a cohort of 60 human mesothelioma samples. Spearman’s correlation.

The observation that the double miR-30 mutant construct (pmiRGLO-CALB2-3′UTR-mir30-dmt) abolished the downregulatory effect mediated by *CALB2*-3′UTR, led us to identify the critical miR-30 member conveying this effect. Computational analysis predicted that only the miR-30a/b/c/d/e-5p arm is assumed to target *CALB2* mRNA. Quantitative expression analysis showed that miR-30b/c/d/e (5p-arm) are abundantly expressed in ZL55, ONE58, ACC-MESO-4 cells (**Figure 4A**). Interestingly, unlike the other miR-30 members where the 3p arm was almost absent, both the miR-30e-5p and -3p arms were expressed in ZL55, ONE58, ACC-MESO-4 cells. miRNA-5p and -3p arms originate from the same pre-miRNA but have a different sequence. Both could become functional miRNA either targeting different mRNA or accomplish a synergistic effect on the same mRNA, but at different sites (Mitra et al., 2015).

ONE58 cells stably expressing Firefly luciferase-CALB2-3′UTR, were transiently transfected with 1 and 5 nM of miR-30b/c/e-5p mimetic treatment (Supplementary Figure 4). Of all tested mimics, 5 nM miR-30e-5p lead to ∼60% downregulation of luciferase expression from the CALB2-3′UTR reporter (*p* = 0.0039) when compared with the negative control mimics (**Figure 4B**). Calretinin protein levels were also decreased after addition of miR-30e-5p in a concentration-dependent manner, i.e., a larger effect with 5 nM mimics (**Figure 4B**). Furthermore, anti-miR-mediated miR-30e-5p inhibition caused a moderate but still significant upregulation of the reporter-CALB2-3′UTR as well as an increase of calretinin protein levels (**Figure 4C**). Similar results were also observed in ACC-MESO-4 cells where the treatment with 5 nM of miR-30e-5p mimetic resulted in ∼50% decrease (*p* = 0.0039) of the reporter expression and in calretinin protein expression (Supplementary Figure 5).

As we demonstrated that the miR-30e-5p was capable to directly regulate calretinin expression, we investigated whether miR-30e-5p expression correlated with calretinin expression, determined by immunohistochemistry scoring of mesothelioma tissue samples. In a pool of 60 mesothelioma tumor samples, calretinin expression versus miR-30e-5p expression showed a weak, but significant negative correlation (**Figure 4D**). Altogether, these findings demonstrated an involvement of miR-30e-5p in negatively regulating calretinin expression.

### Calretinin 3′UTR Acts as Competitive Endogenous RNA

**FIGURE 5:**
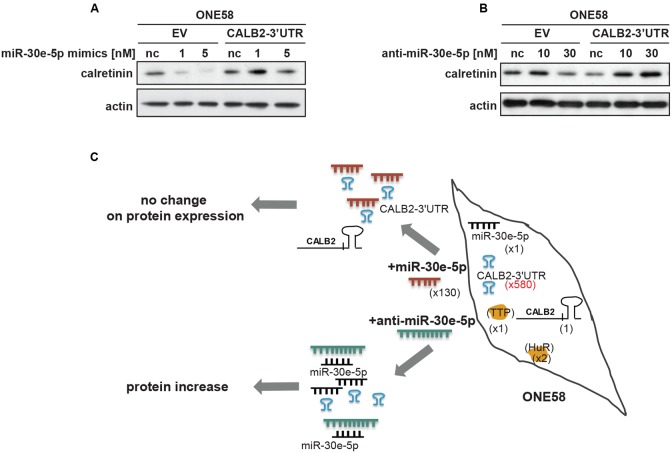
Overexpression of calretinin 3′UTR competes with *trans*-regulatory factors and reveals a possible ceRNA network in regulation of calretinin expression. **(A)** Exogenous overexpression of *CALB2*-3′UTR abolishes the effect of miR-30e-5p on calretinin expression in ONE58 cells. **(B)** Inhibition of miR-30e-5p using anti-miR-30e-5p increased calretinin expression in ONE58 cells stably overexpressing *CALB2*-3′UTR. **(C)** Model of ceRNA network created by introducing exogenous *CALB2*-3′UTR in ONE58 cell. ONE58 cell overexpress 580-times more of calretinin 3′UTR (blue) compared to endogenous calretinin mRNA and twofold more of HuR than TTP (orange). Upon miR-30-5p mimetic treatment (red) no change is observed on calretinin protein levels due to exogenous *CALB2*-3′UTR that sponges miR-30e-5p. The effect of anti-miR-30e-5p (green) is observed in ONE58 cells with exogenous *CALB2*-3′UTR.

According to the so called ceRNA (competitive endogenous RNA) hypothesis, all types of RNA transcripts (coding and non-coding) carrying the same MRE potentially compete for the same miRNA pool therefore acting as natural miRNA sponges (Salmena et al., 2011). Beside miRNA, transcripts may also compete for binding to different RBPs adding yet another level of complexity (Tay et al., 2014). When ONE58 cells stably transfected with empty vector (ONE58-EV-pmiRGLO) or the CALB2-3′UTR construct (ONE58-CALB2-3′UTR) were transiently transfected with miR-30e-5p (∼130-fold more abundant compared to the endogenous miR-30e-5p pool), downregulation of calretinin expression was observed only in ONE58 empty vector expressing cells, suggesting that the constant exogenous overexpression of *CALB2*-3′UTR could sequester miR-30e-5p mimics (**Figure 5A**). Indeed, in ONE58 cell line stably overexpressing the *CALB2*-3′UTR, exogenous *CALB2*-3′UTR levels are ∼580-fold higher (estimated by *Firefly luciferase* vs. *CALB2* mRNA) compared to the endogenous *CALB2* mRNA level. In contrast, when anti-miR-30e-5p was transiently delivered, increase of calretinin protein levels was observed only in ONE58-CALB2-3′UTR and not in ONE58-EV-pmiRGLO cells (**Figure 5B**). Based on our data, we suggest a model in which the *CALB2*-3′UTR might act as a competitor via sequestration of miR-30e-5p thereby decreasing its interaction with the protein coding *CALB2* mRNA isoform (**Figure 5C**). Indeed, ONE58 cells only express low amounts of the full-length isoform 29 kDa calretinin (CALB2) and not the splice variants. In the situation of stable and strong overexpression of CALB2-3′UTR in ONE58 cells, delivery of miR-30e-5p mimics does not repress the expression of calretinin most likely due to the exogenous CALB2-3′UTRs which compete to bind the delivered miR-30e-5p and thus possibly acts as a molecular sponge. In contrast, when the same ONE58-CALB2-3′UTR cells were transfected with anti-miR-30e-5p, calretinin expression was increased (but not in ONE58-empty vector expressing cells), most likely due to repression of miR-30e-5p, which was exerted by both, the presence of exogenous CALB2-3′UTR and anti-miR-30e-5p treatment.

## Discussion

In a previous study we demonstrated a strong positive correlation between calretinin mRNA and protein levels in mesothelioma cell lines (Kresoja-Rakic et al., 2016) and demonstrated NRF-1 and E2F2 to act as transcription factors regulating calretinin expression. In this study, we determined that calretinin expression is additionally regulated in mesothelioma cells through a posttranscriptional mechanism mediated by the 3′UTR. We determined that the CALB2-3′UTR contains two functional elements—a stabilizing ARE motif, and two destabilizing miR-binding sites; the identified miR-30e-5p site was shown to negatively regulate calretinin expression. Moreover, we propose a physiological relevance for the *CALB2* alternative spliced transcripts by their function within a ceRNA network.

3′UTRs play a functional role in the posttranscriptional regulation of mRNA, as the 3′UTR can modulate stability, drive subcellular localization and control translational efficiency of mRNA (Mayr, 2016). Since the 3′UTRs of all three *CALB2* transcripts contain a common 573-nt stretch, we used this region for further functional analysis. Employing a reporter plasmid assay, where the 573-nt stretch of the CALB2-3′UTR was inserted downstream of the Firefly luciferase reporter, we observed that CALB2-3′UTR affected the reporter mRNA stability, as it downregulated the reporter expression in mesothelioma cells.

mRNA stability and therefore mRNA abundance in a cell is controlled by the best studied ARE *cis*-acting elements and by miRNA-binding sites. The core of the ARE sequence is the pentameric motif AUUUA. The ARE motifs exert their function (stabilization or destabilization) by interaction with different ARE-binding proteins (AUBP). Since the mutational approach revealed a stabilization element in the ARE site and we detected HuR, an mRNA stabilizing protein (Brennan and Steitz, 2001), in the ARE-sequence pull-down we propose that HuR is a potential CALB2 regulator. There are several mechanisms through which HuR exerts its stabilization effect. For example, it has been proposed that HuR confers stabilization to mRNA by its ability to outcompete with either destabilizing AUBP (Tiedje et al., 2012) or miRNAs (Bhattacharyya et al., 2006; Srikantan et al., 2012). It would be interesting to test whether silencing HuR in cells expressing CALB2-3′UTR would increase the effects of miR-30e on reporter expression. HuR has been reported to be overexpressed in mesothelioma and HuR cytoplasmic expression significantly correlated with a poor outcome in mesothelioma patients (Stoppoloni et al., 2008). Since a decrease, and not an increase, in calretinin expression is associated with the poorest overall survival, this may suggest that, if it is indeed HuR that stabilizes calretinin mRNA, it competes to bind other tumor progression-associated targets. In fact, in mesothelioma, HuR cytoplasmic expression is correlated with Cyclooxygenase-2 (COX-2) (Stoppoloni et al., 2008), a gene that bears 14 ARE (Sheng et al., 2000). In our panel of six mesothelioma cell lines and by data mining, we observed that HuR was highly expressed and more abundant compared to TTP (destabilizer), supporting that HuR might potentially outcompete the binding of TTP resulting in stabilization of *CALB2* mRNA, since both are able to bind to the same ARE sequence.

The second *cis*-acting element that we found to be functional within CALB2-3′UTR are destabilizing miRNA-binding sites. *CALB2* mRNA was found to interact with Argonaute-1 (AGO1), a protein that is the part of the RNA-induced silencing complex (RISC), which executes the function of miRNA (Li et al., 2014; Jha et al., 2015) indicating that calretinin transcripts are indeed regulated by miRNA. Utilizing several prediction algorithms, miR-30 family members were identified as potential candidates targeting calretinin mRNA; overexpression or inhibition experiments revealed that only miR-30e-5p is able to modulate calretinin protein levels through the calretinin 3′UTR. The miR-30 family includes five members, miR-30a through miR-30e and is evolutionary well conserved. In mesothelioma, high expression of miR-30e-5p has been associated with the epithelioid histopathological subtype, which is generally associated with better prognosis (Busacca et al., 2010). However, upregulation of miR-30e-5p along with other miRNA forms the six-miRNA signature that predicts poorest survival of MPM patients (Kirschner et al., 2015) and this fits with low calretinin levels in epithelioid histotype patients associated with worse outcome (Kao et al., 2011; Otterstrom et al., 2014). Data mining TCGA database revealed that miR-30e-3p and -5p are significantly upregulated in cluster 1, which one of the three clusters obtained based on a consensus non-negative matrix factorization (NMF) clustering method of the 647 most variable miRs (Broad Institute TCGA Genome Data Analysis Center, 2016). In the same cluster miR-31-5p is significantly downregulated consistent with signature findings (Kirschner et al., 2015).

The identification of both positive and negative regulatory elements within CALB2-3′UTR suggests a possible interplay between AUBP and miRNA, adding another level of complexity in regulating calretinin expression, which would help understanding why even in epithelioid MPM not all cells are calretinin-positive (Kao et al., 2011). Different RNA-binding factors can target the same mRNA at different sites or competing for the same site, in a cooperative or antagonistic fashion that depends on their expression levels, localization in the cells or binding affinity (Vislovukh et al., 2014). Various miRNAs may act together with AUBP on the same 3′UTR and may cause reduced RNA stability and/or translation (Jing et al., 2005). For instance, it has been described that the RBP Pumilio-1 (PUM1) alters the secondary structure of target mRNA and thus allowing miR-221 and miR-22 to access the target sites (Kedde et al., 2010). In another example, Dicer and Argonaut (recruited by miR-16) along with TTP are required for ARE-mediated mRNA decoy of tumor necrosis factor alpha (TNFα) (Jing et al., 2005).

The presence of the non-coding alternative spliced calretinin transcripts suggests a potential physiological role of these transcripts, exerted through their 3′UTR. Alternative mRNA calretinin transcripts (CALB2b and CALB2c) had been previously reported in different colon carcinoma cell lines (Schwaller et al., 1995) and in mesenchymal tissue of rat embryos (Schwaller et al., 1995). Almost all multi-exon genes can undergo alternative splicing (Pan et al., 2008). Alternative splicing is a tightly regulated process that contributes to transcriptome and proteome diversity, and has been suggested to be important in developmental stages (Revil et al., 2010). Relatively high abundance of all three transcripts was detected in ACC-MESO-4 cells (high calretinin protein levels), whereas only low abundance of the normally spliced 29-kDa isoform was detected in ONE58 cells (low calretinin protein levels). The same profile of CALB2 transcripts expression was observed in tumors, indicating that alternative splicing is not a cell culture artifact. We suggest that the expression of the alternative transcripts may be useful to protect the full-length transcript from 3′UTR mediated degradation. Indeed, when using exogenous expression of CALB2-3′UTR, we increased ∼580-fold the 3′UTR sequence compared to the endogenous *CALB2* transcripts, therefore likely contributing to the modulation of miR-30e-5p or anti-miR-30e-5p treatment effect in ONE58 cells. This observation led to the hypothesis that in physiological conditions, *CALB2* alternative transcripts might have their role exerted through their 3′UTR by creating a ceRNA network in which they compete to bind miR-30e-5p protecting the protein-coding *CALB2* transcript. Along this line of thinking, it would be interesting to investigate whether other genes sharing the same miR-30e binding site are protected from miRNA-30e-driven downregulation and our data open a new avenue of investigation in that direction. Altogether, our study is providing evidence for the importance and the functionality of the *CALB2*-3′UTR in regulating calretinin expression. Indeed, our data show the role of (1) AUBPs in calretinin stabilization and (2) miR-30e-5p in the posttranscriptional negative regulation of calretinin expression via interaction with its 3′UTR. These results may provide a rationale why targeting 3′UTR sequence for silencing calretinin, a suggested therapeutic approach for epithelioid MPM, is more efficient compared to targeting translated regions (Blum et al., 2017).

Furthermore, our study demonstrates a possible physiological role of calretinin’s alternatively spliced transcripts.

## Author Contributions

JK-R, MS, and MR carried out experiments and interpreted data. EF-B and JK-R designed experiments and interpreted data. MK, SK, and GR provided data on the relationship between CALB2 and miR-30e in clinical samples. JK-R and MK generated figures and tables. JK-R and EF-B wrote the manuscript. All authors read and approved the final manuscript.

## Conflict of Interest Statement

The authors declare that the research was conducted in the absence of any commercial or financial relationships that could be construed as a potential conflict of interest.

## References

[B1] Agarwal V. Bell G. W. Nam J. W. Bartel D. P. (2015). Predicting effective microRNA target sites in mammalian mRNAs. *Elife* 4:e05005 10.7554/eLife.05005 PMC4532895 26267216

[B2] Andre M. Felley-Bosco E. (2003). Heme oxygenase-1 induction by endogenous nitric oxide: influence of intracellular glutathione. *FEBS Lett.* 546 223–227. 10.1016/S0014-5793(03)00576-3 12832044

[B3] Barreau C. Paillard L. Osborne H. B. (2005). AU-rich elements and associated factors: Are there unifying principles? *Nucleic Acids Res.* 33 7138–7150. 10.1093/nar/gki1012 16391004 PMC1325018

[B4] Betel D. Wilson M. Gabow A. Marks D. S. Sander C. (2008). The microRNA.org resource: targets and expression. *Nucleic Acids Res.* 36 D149–D153. 10.1093/nar/gkm995 18158296 PMC2238905

[B5] Bhattacharyya S. N. Habermacher R. Martine U. Closs E. I. Filipowicz W. (2006). Relief of microRNA-mediated translational repression in human cells subjected to stress. *Cell* 125 1111–1124. 10.1016/j.cell.2006.04.031 16777601

[B6] Blum W. Pecze L. Felley-Bosco E. Wu L. de Perrot M. Schwaller B. (2017). Stem cell factor-based identification and functional properties of in vitro-selected subpopulations of malignant mesothelioma cells. *Stem Cell Rep.* 8 1005–1017. 10.1016/j.stemcr.2017.02.005 PMC5390099 28285878

[B7] Blum W. Schwaller B. (2013). Calretinin is essential for mesothelioma cell growth/survival in vitro: a potential new target for malignant mesothelioma therapy? *Int. J. Cancer* 133 2077–2088. 10.1002/ijc.28218 23595591

[B8] Brennan C. M. Steitz J. A. (2001). HuR and mRNA stability. *Cell Mol. Life Sci.* 58 266–277. 10.1007/PL00000854 11289308 PMC11146503

[B9] Broad Institute TCGA Genome Data Analysis Center (2016). *Clustering of miRseq Mature Expression: Consensus NMF. Broad Institute of MIT and Harvard*. Available at: http://gdac.broadinstitute.org/runs/analyses__latest/reports/cancer/MESO-TP/miRseq_Mature_Clustering_CNMF/nozzle.html

[B10] Busacca S. Germano S. De Cecco L. Rinaldi M. Comoglio F. Favero F. (2010). MicroRNA signature of malignant mesothelioma with potential diagnostic and prognostic implications. *Am. J. Respir. Cell Mol. Biol.* 42 312–319. 10.1165/rcmb.2009-0060OC 19502386

[B11] Bustin S. A. Benes V. Garson J. A. Hellemans J. Huggett J. Kubista M. (2009). The MIQE guidelines: minimum information for publication of quantitative real-time PCR experiments. *Clin. Chem.* 55 611–622. 10.1373/clinchem.2008.112797 19246619

[B12] Cheng Y. Y. Wright C. M. Kirschner M. B. Williams M. Sarun K. H. Sytnyk V. (2016). KCa1.1, a calcium-activated potassium channel subunit alpha 1, is targeted by miR-17-5p and modulates cell migration in malignant pleural mesothelioma. *Mol. Cancer* 15:44 10.1186/s12943-016-0529-z PMC4888473 27245839

[B13] Fallmann J. Sedlyarov V. Tanzer A. Kovarik P. Hofacker I. L. (2016). AREsite2: an enhanced database for the comprehensive investigation of AU/GU/U-rich elements. *Nucleic Acids Res.* 44 D90–D95. 10.1093/nar/gkv1238 26602692 PMC4702876

[B14] Fanourgakis G. Lesche M. Akpinar M. Dahl A. Jessberger R. (2016). Chromatoid body protein TDRD6 supports long 3′ UTR triggered nonsense mediated mRNA decay. *PLoS Genet.* 12:e1005857 10.1371/journal.pgen.1005857 PMC4858158 27149095

[B15] Gordon G. J. Rockwell G. N. Jensen R. V. Rheinwald J. G. Glickman J. N. Aronson J. P. (2005). Identification of novel candidate oncogenes and tumor suppressors in malignant pleural mesothelioma using large-scale transcriptional profiling. *Am. J. Pathol.* 166 1827–1840. 10.1016/S0002-9440(10)62492-3 15920167 PMC1363736

[B16] Gruber A. R. Lorenz R. Bernhart S. H. Neubock R. Hofacker I. L. (2008). The Vienna RNA websuite. *Nucleic Acids Res.* 36 W70–W74. 10.1093/nar/gkn188 18424795 PMC2447809

[B17] Hofacker I. L. (2009). RNA secondary structure analysis using the Vienna RNA package. *Curr. Protoc. Bioinformatics* 12.2:12.2.1–12.2.16 10.1002/0471250953.bi1202s26 19496057

[B18] Jha A. Panzade G. Pandey R. Shankar R. (2015). A legion of potential regulatory sRNAs exists beyond the typical microRNAs microcosm. *Nucleic Acids Res.* 43 8713–8724. 10.1093/nar/gkv871 26354861 PMC4605316

[B19] Jing Q. Huang S. Guth S. Zarubin T. Motoyama A. Chen J. (2005). Involvement of microRNA in AU-rich element-mediated mRNA instability. *Cell* 120 623–634. 10.1016/j.cell.2004.12.038 15766526

[B20] Kao S. C. Klebe S. Henderson D. W. Reid G. Chatfield M. Armstrong N. J. (2011). Low calretinin expression and high neutrophil-to-lymphocyte ratio are poor prognostic factors in patients with malignant mesothelioma undergoing extrapleural pneumonectomy. *J. Thorac. Oncol.* 6 1923–1929. 10.1097/JTO.0b013e31822a3740 22011651

[B21] Kedde M. van Kouwenhove M. Zwart W. Oude Vrielink J. A. Elkon R. Agami R. (2010). A Pumilio-induced RNA structure switch in p27-3′ UTR controls miR-221 and miR-222 accessibility. *Nat. Cell Biol.* 12 1014–1020. 10.1038/ncb2105 20818387

[B22] Khabar K. S. (2010). Post-transcriptional control during chronic inflammation and cancer: a focus on AU-rich elements. *Cell Mol. Life Sci.* 67 2937–2955. 10.1007/s00018-010-0383-x 20495997 PMC2921490

[B23] Kirschner M. B. Cheng Y. Y. Armstrong N. J. Lin R. C. Kao S. C. Linton A. (2015). MiR-score: a novel 6-microRNA signature that predicts survival outcomes in patients with malignant pleural mesothelioma. *Mol. Oncol.* 9 715–726. 10.1016/j.molonc.2014.11.007 25497279 PMC5528709

[B24] Krek A. Grun D. Poy M. N. Wolf R. Rosenberg L. Epstein E. J. (2005). Combinatorial microRNA target predictions. *Nat. Genet.* 37 495–500. 10.1038/ng1536 15806104

[B25] Kresoja-Rakic J. Kapaklikaya E. Ziltener G. Dalcher D. Santoro R. Christensen B. C. (2016). Identification of *cis*- and *trans*-acting elements regulating calretinin expression in mesothelioma cells. *Oncotarget* 7 21272–21286. 10.18632/oncotarget.7114 26848772 PMC5008284

[B26] Krol J. Loedige I. Filipowicz W. (2010). The widespread regulation of microRNA biogenesis, function and decay. *Nat. Rev. Genet.* 11 597–610. 10.1038/nrg2843 20661255

[B27] Li J. H. Liu S. Zhou H. Qu L. H. Yang J. H. (2014). starBase v2.0: decoding miRNA-ceRNA, miRNA-ncRNA and protein-RNA interaction networks from large-scale CLIP-Seq data. *Nucleic Acids Res.* 42 D92–D97. 10.1093/nar/gkt1248 24297251 PMC3964941

[B28] Linton A. Pavlakis N. O’Connell R. Soeberg M. Kao S. Clarke S. (2014). Factors associated with survival in a large series of patients with malignant pleural mesothelioma in New South Wales. *Br. J. Cancer* 111 1860–1869. 10.1038/bjc.2014.478 25188323 PMC4453733

[B29] Lu J. Getz G. Miska E. A. Alvarez-Saavedra E. Lamb J. Peck D. (2005). MicroRNA expression profiles classify human cancers. *Nature* 435 834–838. 10.1038/nature03702 15944708

[B30] Ma W. J. Cheng S. Campbell C. Wright A. Furneaux H. (1996). Cloning and characterization of HuR, a ubiquitously expressed Elav-like protein. *J. Biol. Chem.* 271 8144–8151. 8626503 10.1074/jbc.271.14.8144

[B31] Manning L. S. Whitaker D. Murch A. R. Garlepp M. J. Davis M. R. Musk A. W. (1991). Establishment and characterization of five human malignant mesothelioma cell lines derived from pleural effusions. *Int. J. Cancer* 47 285–290. 10.1002/ijc.2910470219 1703129

[B32] Mayr C. (2016). Evolution and biological roles of alternative 3′UTRs. *Trends Cell Biol.* 26 227–237. 10.1016/j.tcb.2015.10.012 26597575 PMC4955613

[B33] Mitra R. Sun J. Zhao Z. (2015). microRNA regulation in cancer: One arm or two arms? *Int. J. Cancer* 137 1516–1518. 10.1002/ijc.29512 25758934 PMC4497856

[B34] Ordóñez N. G. (2014). Value of calretinin immunostaining in diagnostic pathology: a review and update. *Appl. Immunohistochem. Mol. Morphol.* 22 401–415. 10.1097/PAI.0b013e31829b6fbd 24185118

[B35] Otterstrom C. Soltermann A. Opitz I. Felley-Bosco E. Weder W. Stahel R. A. (2014). CD74: a new prognostic factor for patients with malignant pleural mesothelioma. *Br. J. Cancer* 110 2040–2046. 10.1038/bjc.2014.117 24594996 PMC3992494

[B36] Pan Q. Shai O. Lee L. J. Frey B. J. Blencowe B. J. (2008). Deep surveying of alternative splicing complexity in the human transcriptome by high-throughput sequencing. *Nat. Genet.* 40 1413–1415. 10.1038/ng.259 18978789

[B37] Porpodis K. Zarogoulidis P. Boutsikou E. Papaioannou A. Machairiotis N. Tsakiridis K. (2013). Malignant pleural mesothelioma: current and future perspectives. *J. Thorac. Dis.* 5(Suppl. 4), S397–S406. 10.3978/j.issn.2072-1439.2013.08.08 24102013 PMC3791499

[B38] Reid G. (2015). MicroRNAs in mesothelioma: from tumour suppressors and biomarkers to therapeutic targets. *J. Thorac. Dis.* 7 1031–1040. 10.3978/j.issn.2072-1439.2015.04.56 26150916 PMC4466421

[B39] Revil T. Gaffney D. Dias C. Majewski J. Jerome-Majewska L. A. (2010). Alternative splicing is frequent during early embryonic development in mouse. *BMC Genomics* 11:399 10.1186/1471-2164-11-399 PMC2898759 20573213

[B40] Salmena L. Poliseno L. Tay Y. Kats L. Pandolfi P. P. (2011). A ceRNA hypothesis: the Rosetta Stone of a hidden RNA language? *Cell* 146 353–358. 10.1016/j.cell.2011.07.014 21802130 PMC3235919

[B41] Schmitter D. Lauber B. Fagg B. Stahel R. A. (1992). Hematopoietic growth factors secreted by seven human pleural mesothelioma cell lines: interleukin-6 production as a common feature. *Int. J. Cancer* 51 296–301. 10.1002/ijc.2910510220 1373705

[B42] Schwaller B. Celio M. R. Hunziker W. (1995). Alternative splicing of calretinin mRNA leads to different forms of calretinin. *Eur. J. Biochem.* 230 424–430. 10.1111/j.1432-1033.1995.0424h.x 7607211

[B43] Sheng H. Shao J. Dixon D. A. Williams C. S. Prescott S. M. DuBois R. N. (2000). Transforming growth factor-beta1 enhances Ha-ras-induced expression of cyclooxygenase-2 in intestinal epithelial cells via stabilization of mRNA. *J. Biol. Chem.* 275 6628–6635. 10.1074/jbc.275.9.6628 10692471

[B44] Shi Y. Moura U. Opitz I. Soltermann A. Rehrauer H. Thies S. (2012). Role of hedgehog signaling in malignant pleural mesothelioma. *Clin. Cancer Res.* 18 4646–4656. 10.1158/1078-0432.CCR-12-0599 22733539

[B45] Sidi R. Pasello G. Opitz I. Soltermann A. Tutic M. Rehrauer H. (2011). Induction of senescence markers after neo-adjuvant chemotherapy of malignant pleural mesothelioma and association with clinical outcome: an exploratory analysis. *Eur. J. Cancer* 47 326–332. 10.1016/j.ejca.2010.09.044 21036600

[B46] Srikantan S. Tominaga K. Gorospe M. (2012). Functional interplay between RNA-binding protein HuR and microRNAs. *Curr. Protein Pept. Sci.* 13 372–379. 10.2174/138920312801619394 22708488 PMC3535178

[B47] Stoppoloni D. Cardillo I. Verdina A. Vincenzi B. Menegozzo S. Santini M. (2008). Expression of the embryonic lethal abnormal vision-like protein HuR in human mesothelioma: association with cyclooxygenase-2 and prognosis. *Cancer* 113 2761–2769. 10.1002/cncr.23904 18831511

[B48] Tasic B. Menon V. Nguyen T. N. Kim T. K. Jarsky T. Yao Z. (2016). Adult mouse cortical cell taxonomy revealed by single cell transcriptomics. *Nat. Neurosci.* 19 335–346. 10.1038/nn.4216 26727548 PMC4985242

[B49] Tay Y. Rinn J. Pandolfi P. P. (2014). The multilayered complexity of ceRNA crosstalk and competition. *Nature* 505 344–352. 10.1038/nature12986 24429633 PMC4113481

[B50] Thurneysen C. Opitz I. Kurtz S. Weder W. Stahel R. A. Felley-Bosco E. (2009). Functional inactivation of NF2/merlin in human mesothelioma. *Lung Cancer* 64 140–147. 10.1016/j.lungcan.2008.08.014 18835652

[B51] Tiedje C. Ronkina N. Tehrani M. Dhamija S. Laass K. Holtmann H. (2012). The p38/MK2-driven exchange between tristetraprolin and HuR regulates AU-rich element-dependent translation. *PLoS Genet.* 8:e1002977 10.1371/journal.pgen.1002977 PMC3459988 23028373

[B52] Usami N. Fukui T. Kondo M. Taniguchi T. Yokoyama T. Mori S. (2006). Establishment and characterization of four malignant pleural mesothelioma cell lines from Japanese patients. *Cancer Sci.* 97 387–394. 10.1111/j.1349-7006.2006.00184.x 16630136 PMC11158456

[B53] van Kouwenhove M. Kedde M. Agami R. (2011). MicroRNA regulation by RNA-binding proteins and its implications for cancer. *Nat. Rev. Cancer* 11 644–656. 10.1038/nrc3107 21822212

[B54] Vislovukh A. Vargas T. R. Polesskaya A. Groisman I. (2014). Role of 3′-untranslated region translational control in cancer development, diagnostics and treatment. *World J. Biol. Chem.* 5 40–57. 10.4331/wjbc.v5.i1.40 24600513 PMC3942541

[B55] Volinia S. Calin G. A. Liu C. G. Ambs S. Cimmino A. Petrocca F. (2006). A microRNA expression signature of human solid tumors defines cancer gene targets. *Proc. Natl. Acad. Sci. U.S.A.* 103 2257–2261. 10.1073/pnas.0510565103 16461460 PMC1413718

[B56] von Roretz C. Di Marco S. Mazroui R. Gallouzi I. E. (2011). Turnover of AU-rich-containing mRNAs during stress: a matter of survival. *Wiley Interdiscip. Rev. RNA* 2 336–347. 10.1002/wrna.55 21957021

[B57] von Roretz C. Gallouzi I. E. (2008). Decoding ARE-mediated decay: is microRNA part of the equation? *J. Cell Biol* 181 189–194. 10.1083/jcb.200712054 18411313 PMC2315667

