# Supplementary material for: Posttranscriptional Regulation Controls Calretinin Expression in Malignant Pleural Mesothelioma
Source: Front Genet. 2017 May 29;8:70. doi: 10.3389/fgene.2017.00070 (PMC5447031; doi:10.3389/fgene.2017.00070)
Supplement: Supplementary file 1 [file Data_Sheet_1.DOCX]

Supplementary Material

Posttranscriptional regulation controls calretinin expression in malignant pleural mesothelioma

Jelena Kresoja-Rakic^1^, Merve Sulemani^1^, Michaela B. Kirschner^2^, Manuel Ronner^1^, Glen Reid^3,4^, Steven Kao^3,4,5^, Beat Schwaller^6^, Walter Weder^2^, Rolf A. Stahel^7^, Emanuela Felley-Bosco^1*^

*** Correspondence:** Corresponding Author: [emanuela.felley-bosco@usz.ch](mailto:emanuela.felley-bosco@usz.ch)

# Supplementary Figures and Tables

## Supplementary Figures


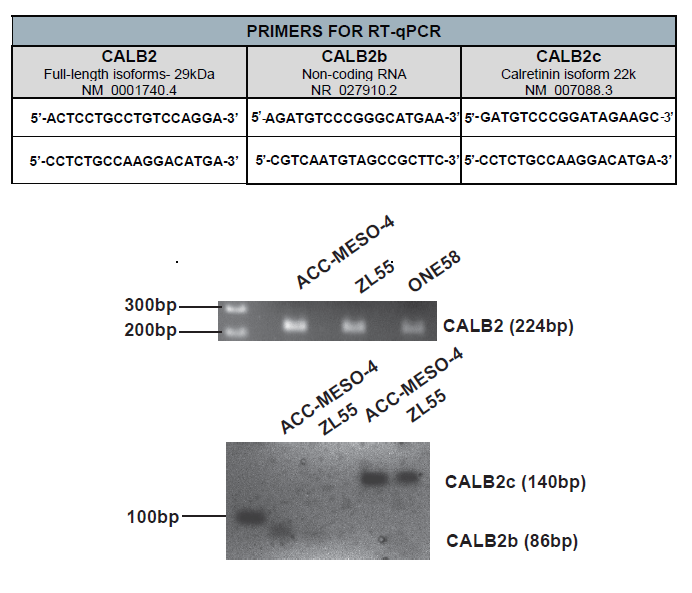


**Supplementary Figure 1** Expression of 3 different calretinin transcripts in mesothelioma cell lines. Primer sequences used to detect different calretinin transcript and analysis of the different products obtained from cDNA of ACC-MESO-4, ZL55 and ONE58 cells.


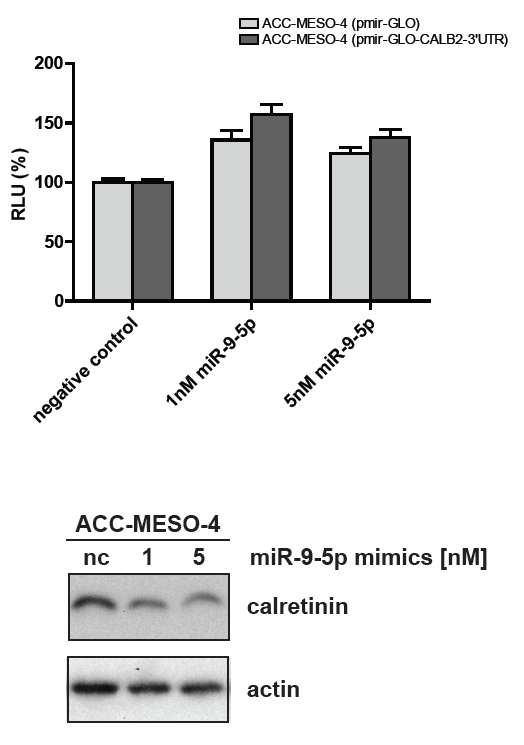


**Supplementary Figure 2** miR-9-5p mimics treatment did not exert downregulatory effect on the reporter expression and calretinin expression in ACC-MESO-4 cells.


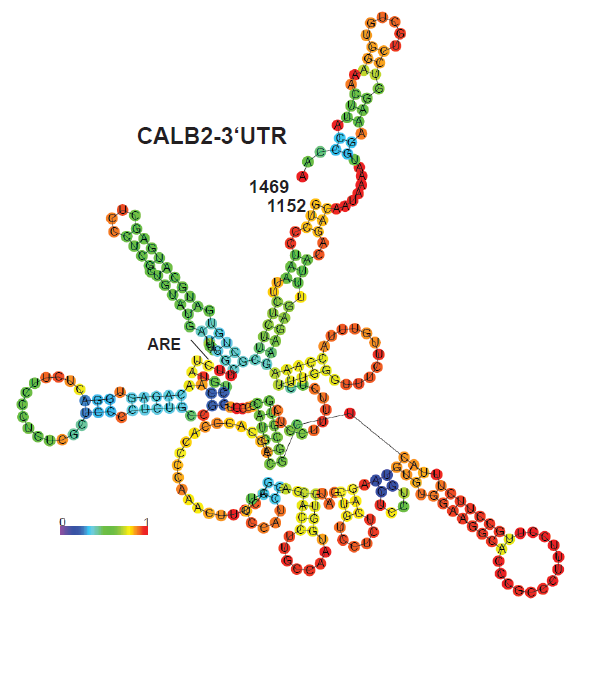


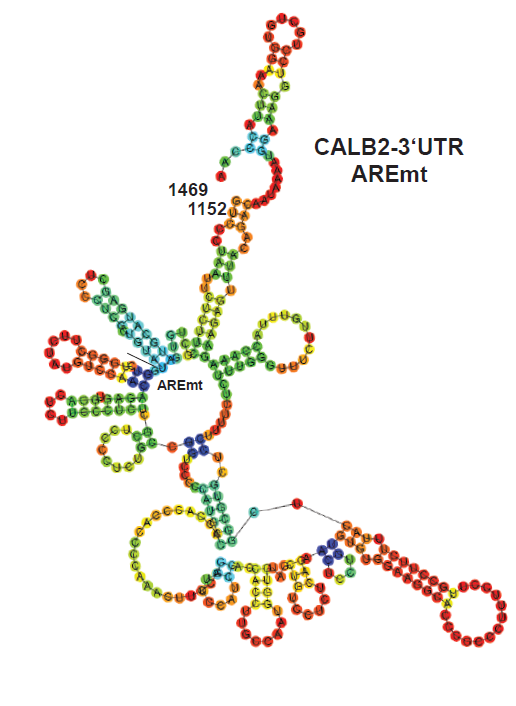


**Supplementary Figure 3** Predicted optimal secondary structures of the wt and mtARE calretinin 3’UTR fragment (317 nt; position 1152-1469) using RNAfold software. The ARE-motif (AUUUA) is forming a bulge, which upon mutation of the consensus sequence is abolished. The colors represent base pair probabilities (0 to 1) for paired- or unpaired bases.


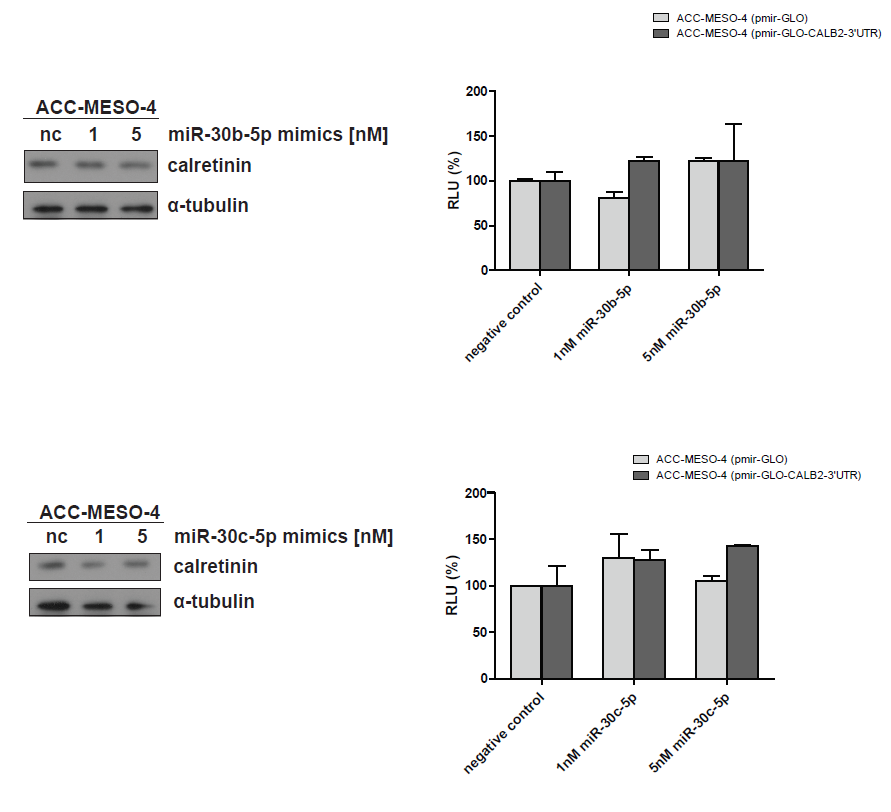


**Supplementary Figure 4** miR-30b/c-5p mimics treatment did not exert downregulatory effect on the reporter expression and calretinin expression in ACC-MESO-4 cells.

**
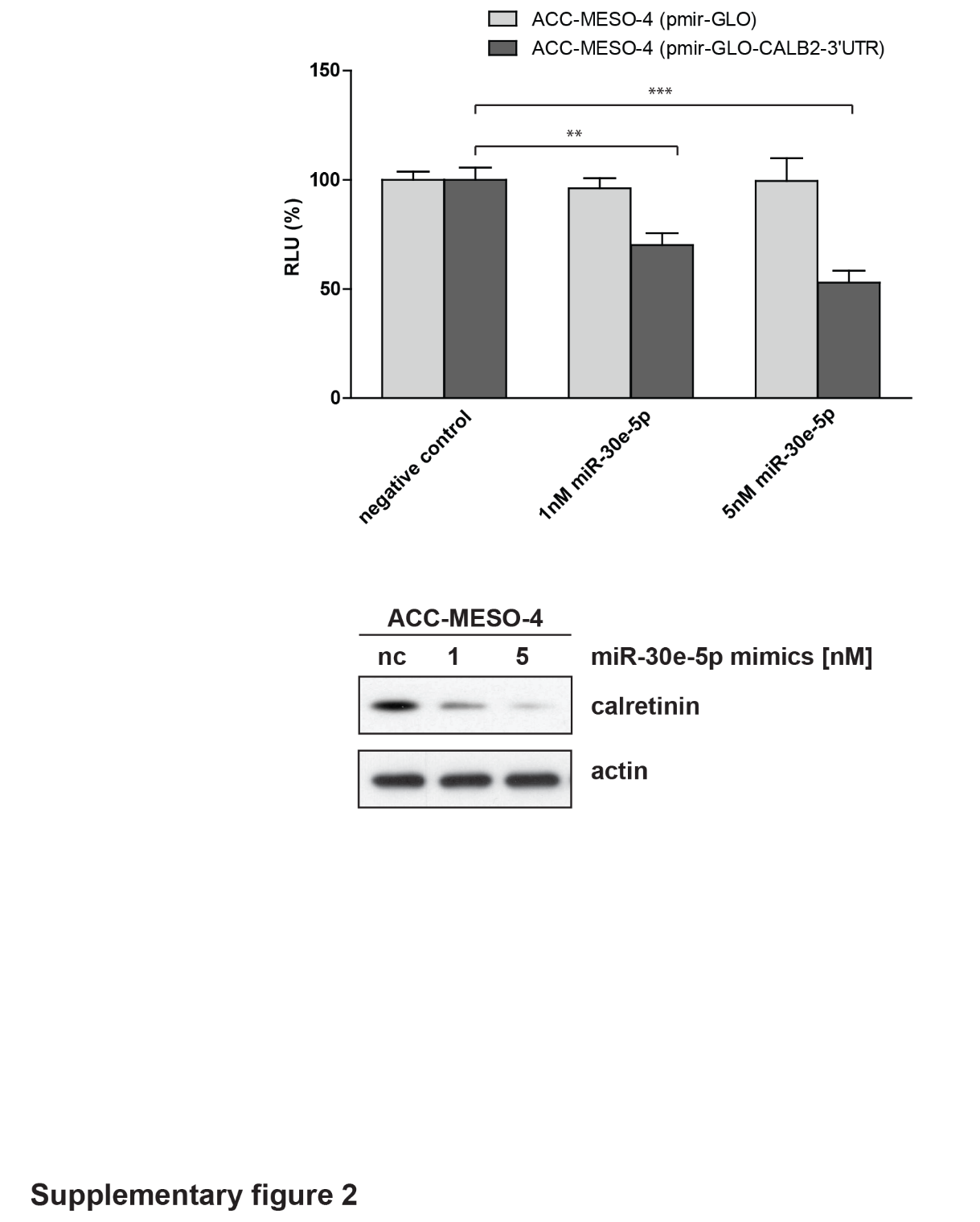
**

**Supplementary** Figure 5 miR-30e-5p regulates calretinin expression also in ACC-MESO-4 cells. miR-30e-5p mimic treatment significantly repressed reporter expression in ACC-MESO-4 cells stably expressing reporter-calretinin 3’UTR along with downregulation of calretinin protein levels. Mean, ±SD n=6; * p<0.05; **p<0.01; ***p<0.005 using Mann-Whitney U test.

## Supplementary Table

**Supplementary Table 1**

Baseline Patient Characteristics (N=60)

| Median Age (Range) | 58 (22 – 74) |
| --- | --- |
| Gender  Male  Female | 46 (76.7 %)  14 (23.3 %) |
| Histological Subtype  Epithelioid  Biphasic  Sarcomatoid | 43 (71.7 %)  17 (28.3 %)  0 (0 %) |
| Pathological Stage  I  II  III  IV | 2 (3.3 %)  8 (13.3 %)  44 (73.3 %)  6 (10.0 %) |
| Induction Chemotherapy  Yes  No | 13 (21.7 %)  47 (78.3 %) |
| Overall survival from surgery (months, range) | 15.3 (0.1 – 90.5) |
